# Advances in *Quercus ilex* L. breeding: the CRISPR/Cas9 technology via ribonucleoproteins

**DOI:** 10.3389/fpls.2024.1323390

**Published:** 2024-02-19

**Authors:** Vera Pavese, Andrea Moglia, Anna Maria Milani, Lorenzo Antonio Marino, Maria Teresa Martinez, Daniela Torello Marinoni, Roberto Botta, Elena Corredoira

**Affiliations:** ^1^ Dipartimento di Scienze Agrarie, Forestali e Alimentari-Department of Agricultural, Forest and Food Sciences (DISAFA), Università degli Studi di Torino, Torino, Italy; ^2^ Mision Biologica de Galicia, Sede de Santiago, Consejo Superior de Investigaciones Cientificas, Santiago de Compostela, Spain

**Keywords:** forest biotechnology, holm oak, gene editing, green fluorescence protein, protoplasts, transgene-free, phytoene desaturase gene

## Abstract

The CRISPR/Cas9 ribonucleoprotein (RNP)-mediated technology represents a fascinating tool for modifying gene expression or mutagenesis as this system allows for obtaining *transgene-free* plants, avoiding exogenous DNA integration. Holm oak (*Quercus ilex*) has an important social, economic, and ecological role in the Mediterranean climate zones of Western Europe and North Africa and is severely affected by oak decline syndrome. Here we report the first example of the application of the CRISPR/Cas9-RNP technology in holm oak. Firstly, we evaluated the protoplast isolation from both *in vitro* leaves and proembryogenic masses. Proembryogenic masses represented the best material to get high protoplast yield (11 x 10^6^ protoplasts/ml) and viability. Secondly, the protoplast transfection ability was evaluated through a vector expressing green fluorescence protein as marker gene of transfection, reaching a transfection percentage of 62% after 24 hours. CRISPR/Cas9 RNPs were successfully delivered into protoplasts resulting in 5.6% ± 0.5% editing efficiency at *phytoene desaturase* (*pds*) target genomic region. Protoplasts were then cultured in semisolid media and, after 45 days in culture, developed embryogenic calli were observed in a Murashige and Skoog media with half concentration of NH_4_NO_3_ and KNO_3_ supplemented with 0.1 mg/L benzylaminopurine and 0.1 mg/L 2,4-dichlorophenoxyacetic acid.

## Introduction

*Dehesas* constitutes the most characteristic type of agrosilvopastoral system in Europe (Pulido et al., 2001), stretching over an area of 5.8 million hectares in Spain and 0.5 million hectares in Portugal (Horrillo et al., 2016). They are artificial ecosystems, in which, thanks to a sustainable management model, marginal land that is unsuitable for growing other crops is converted into biodiverse areas where agricultural, livestock and forest production are combined (Escribano et al., 2022). In the *dehesas*, forage (pasture) crops coexist with dispersed trees, mainly holm oak (*Quercus ilex* L.). This tree is characteristic of the Mediterranean climate and is, in fact, the most abundant tree species in the Iberian Peninsula and other Mediterranean regions. Holm oak is valued for its timber and particularly for its acorns (the fruit of oak trees), which are fed to Iberian pigs, from which nationally and internationally renowned gastronomic products are obtained (Canellas et al., 2007). In addition to being economically valuable, the *dehesas* and holm oaks are also of high ecological and environmental value: they help to protect the soil from erosion, form part of the water cycle, participate in carbon sequestration, protect biodiversity, by providing a habitat for numerous species, and regulate the local climate (Kay et al., 2019).

In recent years, holm oaks have been seriously damaged by oak decline, which is caused by a wide range of biotic and abiotic stressors, thus affecting the viability and sustainability of the *dehesas*. Among these stressors, *Phytophthora cinnamomi* Rands is considered the main pathogen responsible for the oak decline. In addition, the effects of climate change, with increasing periods of drought, aggravate the pathology caused by *P. cinnamomi*, and survival of this agroforestry system is becoming a challenge (Morcillo et al., 2022).

Conventional breeding programs are difficult in woody species as holm oak, due to the long juvenile period, recalcitrance to vegetative propagation and a high heterozygosity degree of the species (Martinez et al., 2019). Defining new strategies for producing holm oak plants with enhanced *P. cinnamomi* tolerance and improved acorn production is thus important to ensure the health and sustainability of the *dehesas*.

Advancements in plant-breeding techniques have enabled much faster production of new plant varieties with desired traits. Among these, the CRISPR/Cas9 (Clustered Regularly Interspaced Short Palindromic Repeats) system represents a real revolution in all areas of biotechnology, enabling the production of ideal cultivars free of negative or undesired genetic traits (Pavese et al., 2021a; Vaia et al., 2022). However, until now, the use of this technique in forest species remains limited (Walawage et al., 2019; Wang et al., 2020; Poovaiah et al., 2021; Pavese et al., 2021a, 2022) due to their recalcitrance to *in vitro* regeneration and the low transformation efficiency (Corredoira et al., 2019). This applies to holm oak, in which although somatic embryogenesis has been induced in zygotic embryos (Mauri and Manzanera, 2003), flower tissues (Blasco et al., 2013; Barra-Jimenez et al., 2014) and leaf and apex explants derived from *in vitro* cultures (Martinez et al., 2017; 2020; 2021), the induction frequency is usually low and the success of the technique is highly genotype dependent. Procedures for *Agrobacterium*-mediated genetic transformation in holm oak have been reported, but the transformation rates are low (less than 2.5%) and long *in vitro* culture periods are required to get regeneration (Cano et al., 2020; Serrazina et al., 2022).

The CRISPR/Cas9 machinery is generally delivered into plant cells using *Agrobacterium tumefaciens* strain. However, this procedure can provoke different negative effects: the vector can be integrated into the host genome and is remain active, the off-target cleavage, and the random insertion of foreign DNA into the genome (Amirkhanov and Stepanov, 2019). To avoid these issues, the CRISPR/Cas9 machinery can be delivered in the ribonucleoprotein (RNP) form which acts on the target gene before being rapidly degraded. This technology represents the new frontier of *gene editing*, promoting the achievement of transgene-free plants (Park and Choe, 2019), thus increasing consumers acceptability (Murovec et al., 2018). The efficiency of this approach has already been tested on several commercial crops (for review, please see Metje-Sprink et al., 2019). By contrast, this technology is poorly explored in woody species and until now few reports are available (Malnoy et al., 2016; Osakabe et al., 2018; Pavese et al., 2022; Vaia et al., 2022).

In view of the high economic relevance of the holm oak in the Mediterranean region and the high incidence of oak decline syndrome, further generation of tolerant material by new breeding tools is needed. The main objectives of the present study were (1) to define a procedure to obtain protoplasts from different types of tissues of holm oak, (2) to test the transfection ability of the protoplasts using the *Green Fluorescence protein* (GFP) and (3) to test the CRISPR/Cas9 ribonucleoprotein (RNP)-mediated technology targeting the *phytoene desaturase* (*pds*) gene, whose knock-out causes the appearance of an albino phenotype.

## Materials and methods

### Plant material and growth conditions

For protoplast isolation, both proembryogenic masses (PEMs) isolated from an embryogenic line of *Q. ilex* and young leaves excised from axillary shoot cultures of *Q. ilex* were used as starting material (**Supplementary Material 1**). The embryogenic line was induced from teguments of ovules derived from adult trees (Barra-Jimenez et al., 2014). This line was maintained by secondary embryogenesis with periodic subcultures, every 6 weeks, on Schenk and Hildebrandt macro and micronutrients [Schenk and Hildebrandt, 1972 (SH)] (Duchefa Biochemie, Netherlands), Murashige and Skoog vitamins (Murashige and Skoog, 1962; MS) (Duchefa Biochemie, Netherlands), 30 g/L sucrose and 6 g/L Plant Propagation Agar (Pronadisa, Spain).

Axillary shoot cultures were established and maintained as described by Martinez et al. (2020). Briefly, for maintenance shoots were cultured on McCown and Lloyd (1981) (WPM) mineral medium (Duchefa Biochemie, Netherlands) supplemented with 3% sucrose, sigma Agar (A-1296; Sigma-Aldrich, St. Louis, MO), and 20 µM silver thiosulphate in an alternating 2 week-long subcultures on 0.44 µM 6-benzyadenine (BA) first, followed by 0.22 µM BA.

Unless specified, all cultures were grown in a photoperiodic climatic chamber with a 16h light and 8h dark photoperiod (standard conditions). Illumination was provided by tubes of white light fluorescent lamps (Maz-dafluor 7D TF 36 w/LJ) with a photon flux density of 50-60 μmol.m^-2^.s^-1^.

### Protoplast isolation

Two different mineral solutions were tested to isolate protoplasts: i) the CPW buffer consisting of 0.2 mM KH_2_PO_4_, 1 mM KNO_3_, 10.1 mM CaCl_2_ x 2H_2_O, 1 mM MgSO_3_ x 7H_2_O, 0.96 μM KI, 0.16 μM CuSO_4_ x 5H_2_O, 11% D-Mannitol, 0.1% BSA (Bovine serum albumine) (pH 5.7) (Kuzminsky et al., 2016) and ii) the NEW mineral solution consisting of 20 mM morpholinoethane sulfonic acid (MES), 0.5 M mannitol, 20 mM KCl, and 10 mM CaCl_2_ (pH 5.7) defined by Pavese et al. (2022).

Both leaves (0.1 g) and proembryogenic masses (0.1 g) were used for protoplast isolation trials to test the most suitable explant in terms of protoplast yield and quality. All the solutions are described in **Supplementary Material 2**. Leaves were dissected as previously mentioned by Kuzminsky et al. (2016). Briefly, after applying a thin layer of polyvinylpyrrolidone (PVP-40) powder to cover the leaf lamina, trichomes were eliminated and leaves were scratched in the CPW washing buffer using a sterile scalpel. Subsequently, leaves were cut into small pieces after removing the midrib region and petiole. Then, different types of enzymes and concentrations were evaluated by adding directly to CPW and NEW solutions: a) 3% Cellulase R-10 (Duchefa Biochemie, Netherlands), plus 1.5% Macerozyme R-10 (Duchefa Biochemie, Netherlands), 1.5% Hemicellulase from *Aspergillus niger* (Sigma-Aldrich, St. Louis, MO); b) 2% Cellulase R-10, 0.75% Macerozyme R-10 and 0.75% Hemicellulase; c) 1% Cellulase R-10, 0.5% Macerozyme R-10 and 0.5% Hemicellulase; d) 3% Cellulase R-10, 1.5% Macerozyme R-10 and 1.5% Pectolyase Y-33 (Duchefa Biochemie, Netherlands); e) 2% Cellulase R-10, 0.75% Macerozyme R-10 and 0.75% Pectolyase Y-33; f) 1% Cellulase R-10, 0.5% Macerozyme R-10 and 0.5% Pectolyase Y-33.

For PEMs, 1.5% Cellulase R-10 and 0.5% Macerozyme R-10 were applied as previously reported with high protoplast isolation by Pavese et al. (2022). PEMs were dissected in small clumps before adding the enzymatic mixture.

To activate enzymes, both cut explants were treated with enzyme solution preheated at 55°C. After, explants were subjected to 20 minutes of vacuum infiltration, followed by maintenance on the rotary shaker in dark conditions (37°C at 40 rpm) (Pavese et al., 2022). Leaves were kept in agitation overnight and protoplast yield was evaluated after 10h and 12h from the beginning of the digestion process. PEMs were subjected to a short period of digestion compared to leaves explants and protoplast yield was evaluated from 4h to 6h following Pavese et al. (2022).

Protoplast solution was then filtered through a 100 μM nylon mesh to eliminate cell wall debris and an equal volume of washing solution (WS) was added to guarantee the right osmolarity. The protoplast suspension was then centrifuged at 300 g for 10 minutes, removing the supernatant and the pellet was resuspended in 5 mL of WS. Protoplasts were then transferred on a 21% (w/v) sucrose gradient, and after centrifugation (300 g for 5 minutes) the ring of viable undamaged protoplasts was aspirated and resuspended in 1 mL of WS. Protoplasts were cleaned two times using WS and then resuspended in 300 µL MMG solution to maintain the osmolarity as described by Osakabe et al. (2018).

The protoplast yield was evaluated using the hemocytometer whereas the viability test was performed using the Trypan blue staining at 4% (w/v) (Sigma-Aldrich, St. Louis, MO) using the B-190 Series OPTIKA microscope, Italy. The percentage of viable protoplasts was defined as the number of viable protoplasts (i.e. not coloured in blue) per the total number of observed protoplasts x 100%. Protoplasts were then diluted in 100 µL of MMG to achieve a final concentration of 2 × 10^5^ and maintained overnight at 4°C.

**Figure 1:**
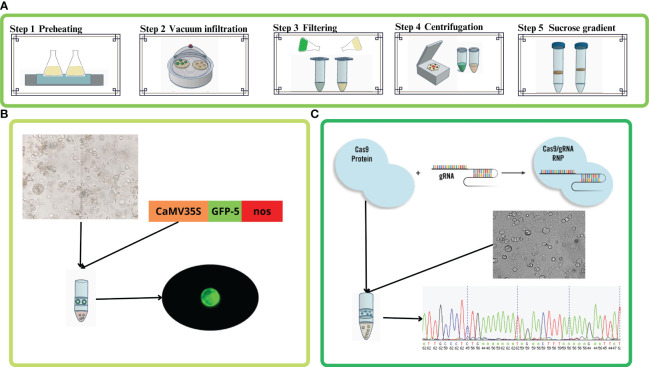
Setting up of CRISPR/Cas9 ribonucleoprotein (RNP) genome editing in holm oak. **(A)** Protocol for protoplast isolation from *in vitro* leaves (green) and proembryogenic masses (yellow). Step 1: enzymatic solution preheating. Step 2: enzymatic digestion and vacuum infiltration. Step 3: filtering and debris removal. Step 4: centrifugation for protoplast concentration and cleaning. Step 5: sucrose gradient and cleaning. Step 6: microscopic observation and viability test. **(B)** Protoplast transfection using the pAVA393:GFP plasmid constituted of 35S promoter, GFP gene and the T-nos terminator. The GFP fluorescence was evaluated under the fluorescence microscope. **(C)** Schematic representation of protoplast transfection using RNPs. Transfection process followed by DNA extraction, PCR amplification, and Sanger sequencing followed by chromatogram analysis.

A schematic overview of the experimental design is available in **Figure 1A** and **Supplementary Material 3**.

### Protoplast transfection using the GFP plasmid

The plasmid pAVA393 (Ochatt et al., 2005) containing the GFP marker gene was used for the first transfection trial (**Figure 1B**). The 100 µL of diluted protoplasts were used and three biological replicates were performed to guarantee statistical uniformity. Protoplasts were carefully mixed with 10 or 20 μg of pAVA393:GFP plasmid DNA and then 100 μL of 40% (w/v) polyethylene glycol (PEG) was added. The mixture was incubated for 20 minutes at room temperature followed by two rinses in 1 mL of WS. Protoplasts were maintained at room temperature in the dark until the evaluation of the transfection efficiency at 12h, 24h, and 48h using the fluorescence microscope (Nikon Eclipse Ti2, Japan). The GFP emission was examined at 516 nm and the transfection efficiency was defined as the percentage of fluorescent cells per the total number of observed cells. Statistical analysis was performed by one-way factorial ANOVA (ANOVA I) applying SPSS software for Windows (version 27.0, SPSS Inc., Chicago, IL, USA). Protoplast DNA was extracted from all the experimental time points using the E.Z.N.A.^®^ Plant DNA kit (Omega Bio-tek, Norcross, GA, USA). PCR was performed using pAVA393:GFP specific primers (F-5CACGACGTTGTAAAACGAC3; R-5GGATAACAATTTCACACAGG3) and *Cm7-actin* housekeeping, previously reported by Pavese et al., 2021b. The amplification conditions were: 95°C/3 min, 30 cycles at 95°C/30 s, 58°C/30 s, 72°C/45 s, followed by an elongation step at 72°C/5 min.

### Protoplast transfection via Cas9 ribonucleoprotein

The gene delivery for the first protoplast transfection event using RNP was the *phytoene desaturase* gene (*pds*), previously identified in *Castanea sativa* by Pavese et al. (2021a) (**Supplementary Material 4**). In particular, we tested if the sgRNA targeting *C* ***spds*** previously designed (5GAGTCAAGAGATGTGCTAGG 3) (Pavese et al., 2022) could target the holm oak *pds*, due to the sequence homology.

DNA was extracted from both leaves (0.1g) and PEMs (0.1g) of holm oak, by EZNA Plant DNA kit (Omega Bio-tek, Norcross, GA, USA). The PCR of the *pds* genomic region was carried out using KAPA HIFI Taq (Roche, Mannheim, Germany) and the *C. sativa* primers designed by Pavese et al., 2022, (Seq_*pds*_F : TGGAAACTTTGGGTATGCATCC; Seq_*pds*_R: TTCTGTGATTGGTAGGCTTTCA). The amplification conditions were: 95°C/3 min, 30 cycles at 98°C/20 s, 60°C/20 s, 72°C/45 s, followed by an elongation step at 72°C/3 min.

PCR products were then purified through DNA/RNA Clean Up E.Z.N.A.^®^ kit (Omega Bio-tek, Norcross, GA, USA) and sequenced by Sanger method. MEGAX alignment between *Q. ilex* and *C. sativa pds* region was carried out to evaluate the homology degree.

Once the *pds* sequence homology between oak and chestnut was verified (**Figure 1C**), the protoplast transfection via Cas9 RNPs was performed according to Pavese et al., 2022. Three biological replicates of protoplast DNA were extracted using the EZNA Plant DNA kit (Omega Bio-tek, Norcross, GA, USA). Then PCR reaction was performed and products were purified through DNA/RNA Clean Up E.Z.N.A.^®^ kit (Omega Bio-tek, Norcross, GA, USA) and sequenced. According to the previously reported work on plant protoplasts (Poovaiah et al., 2021; Pavese et al., 2022; Scintilla et al., 2021; Brandt et al., 2020) for the screening protocol, the Sanger method was used as a cost-effective and less time-consuming sequencing platform compared to high-throughput sequencing methods (Lin et al., 2018; Sant'Ana et al., 2020). Moreover, the Sanger sequencing method allows to obtain direct and detailed information on the mutation frequencies and types (Liang et al., 2018). The chromatograms were then analyzed through the bioinformatic software TIDE (Tracking of Indels by Decomposition) (https://tide.deskgen.com, accessed on 08/March/2023), a simple and accurate tool to determine the typology and frequency of targeted mutations in a cell pool. In a previous work the editing efficiency predicted by the Tracking of Indels by Decomposition (TIDE) assay was compared to that observed by targeted NGS for cellular pools. It has been shown that targeted NGS and TIDE assays predict similar editing efficiencies for pools of cells (Sentmanat et al., 2018).

Transfected protoplasts were compared to three untreated controls samples and three samples treated only with gRNA without the addition of the Cas9 nuclease.

### Protoplasts regeneration

Protoplasts were incubated in three different regenerations media named Q1, Q2 and Q3 using semi-solid agar disc-culture method (**Supplementary Material 2**). Protoplasts were incorporated in semi-solid media surrounded by an identical medium deprived of agar. Protoplast cultures were incubated at 24°C in dark. Fresh medium was replaced weekly. Protoplast growth was monitored through microscopic analysis (Leica-Wild Heerbrugg M8 stereoscope (Leica, Germany)).

### Statistical analysis

Data were statistically elaborated by one-way ANOVA using the software SPSS for Windows (version 28.0, SPSS Inc., Chicago, IL, USA). Different letters associated with the set of means indicate a significant difference based on Tukeys HSD test (p ≤ 0.05).

## Results

### Protoplasts isolation from leaves

The isolation of protoplasts from *in vitro* leaves of holm oak was performed using six combinations and concentrations of enzymes and the release of protoplasts was evaluated at 10h and 12h from the beginning of the enzymatic action both in CPW (Kuzminsky et al., 2016) and in NEW mineral solutions (Pavese et al., 2022).

**Figure 2:**
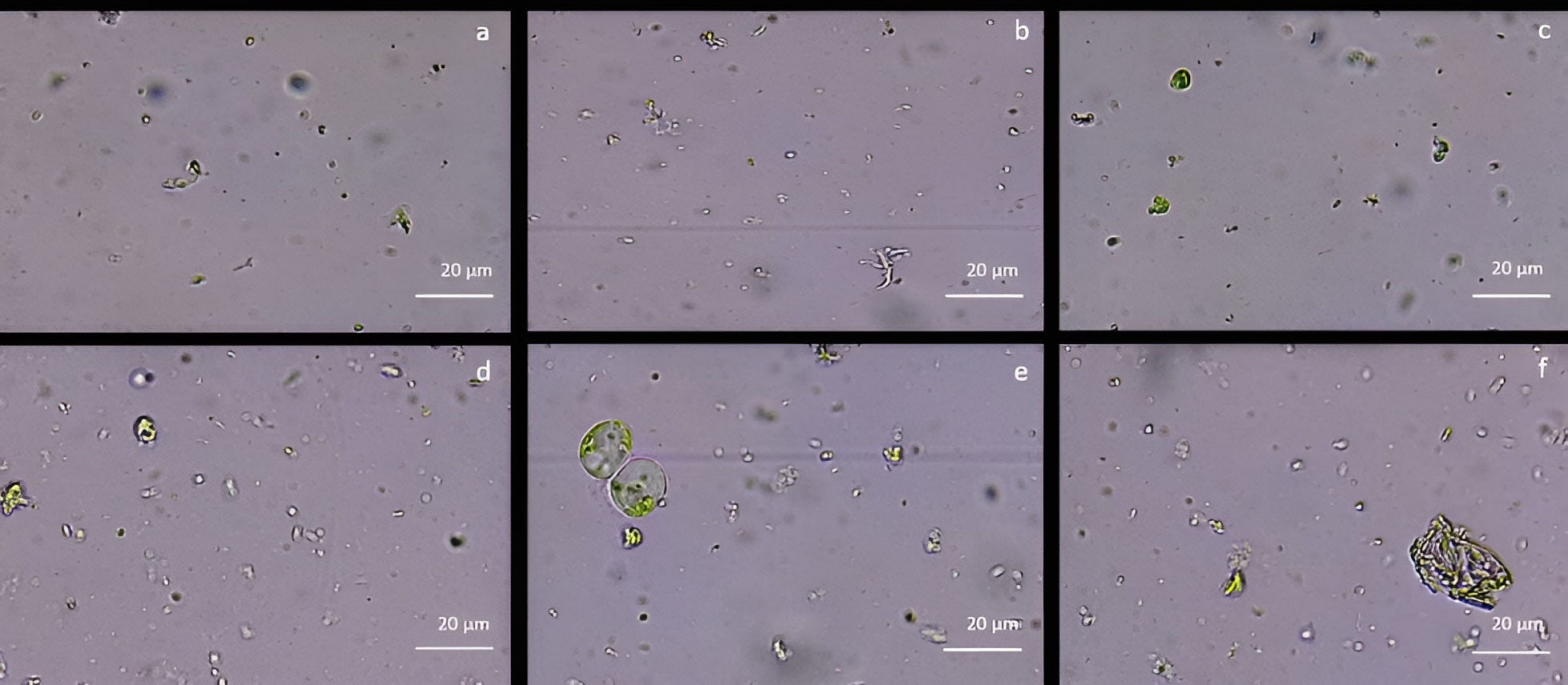
Protoplast visualization at 12h from holm oak leaf digestion in CPW mineral salts supplemented with different enzyme types and concentrations **(A)** 3% Cellulase R-10, 1.5% Macerozyme R-10 and 1.5% Hemicellulase; **(B)** 2% Cellulase R-10, 0.75% Macerozyme R-10 and 0.75% Hemicellulase; **(C)** 1% Cellulase R-10, 0.5% Macerozyme R-10 and 0.5% Hemicellulase; **(D)** 3% Cellulase R-10, 1.5% Macerozyme R-10 and 1.5% Pectolyase Y-33; **(E)** 2% Cellulase, 0.75% Macerozyme R-10 and 0.75% Pectolyase Y-33; **(F)** 1% Cellulase R-10, 0.5% Macerozyme R-10 and 0.5% Pectolyase Y-33. Protoplasts are highlighted by the red arrows. Bar: 20µm; Magnification 40X.

As reported in **Figure 2**, cell wall digestion occurred only after 12h in CPW mineral solution treatment with 2% Cellulase R-10, 0.75% Macerozyme R-10 and 0.75% Pectolyase Y-33 (**Figure 2E**). Under these conditions, the protoplast yield, evaluated using the hemocytometer, was 1,2 x 10^6^ ± 0.3 x 10^6^ protoplasts/mL with 82% ± 1 viability. In all the other tested enzyme conditions, only debris and intact cells were detected (**Figure 2**). By using NEW mineral solution, no evidence of protoplast release was detected in all the enzymatic conditions tested both at 10 and 12h after the enzymatic treatment.

### Protoplasts isolation from proembryogenic masses

**Figure 3:**
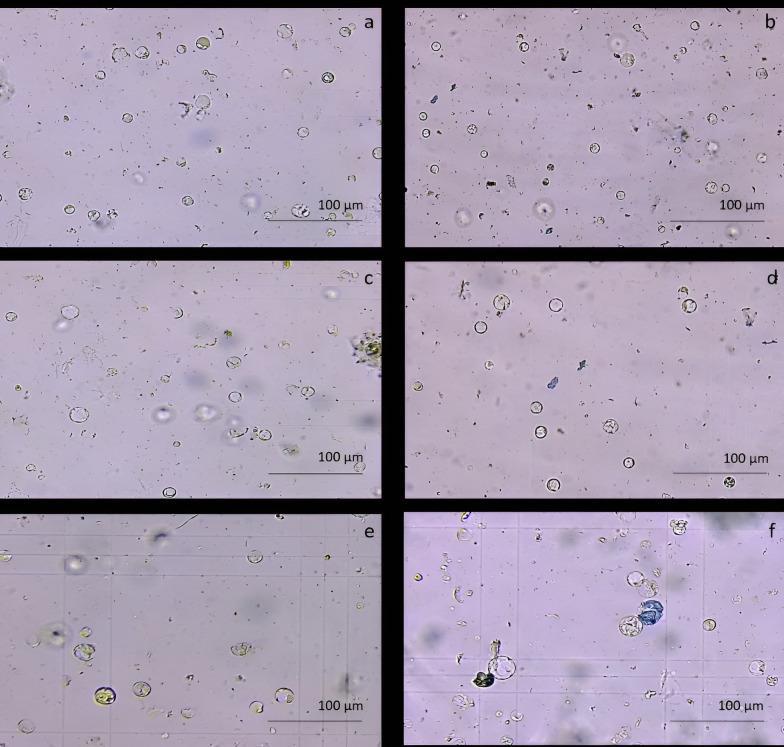
Comparison of protoplast isolation protocols using proembryogenic masses and leaves of holm oak. **(A-D)** Protoplast visualization at 6h from proembryogenic masses of holm oak; a) Protoplasts isolated in NEW mineral solution; b) viability test on protoplasts isolated in NEW mineral solution; **(C)** protoplasts isolated in CPW mineral solution; **(D)** viability test on protoplasts isolated in CPW mineral solution; **(E, F)** Protoplast visualization at 12h from leaves of holm oak; **(E)** protoplasts isolated from leaves in CPW mineral solution; f) viability test on protoplasts isolated from leaves in CPW mineral solution. Bar: 100 µm, Magnification 10X.

**Figure 4:**
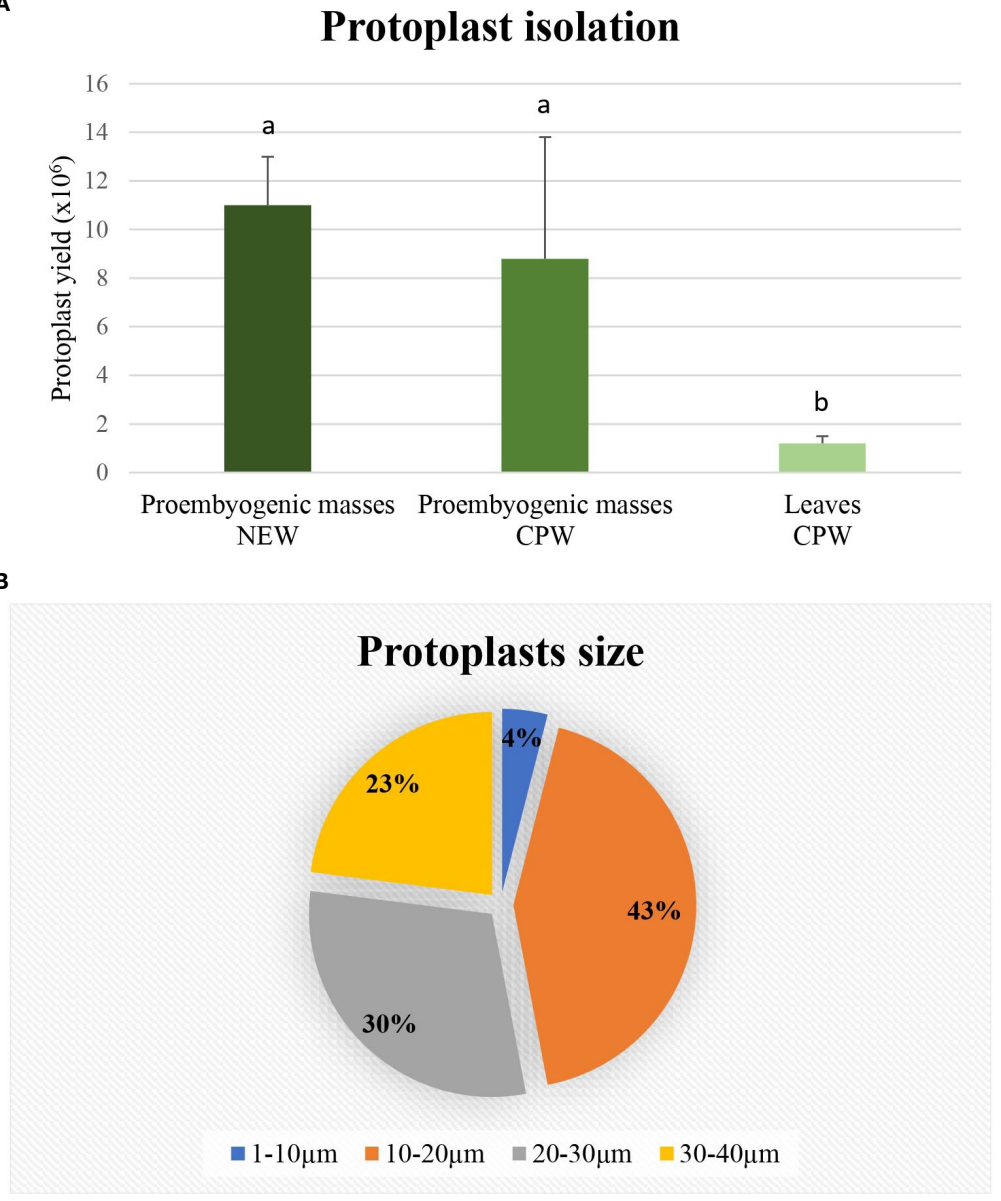
Determination of protoplasts production and protoplast size. **(A)** Yield comparison between protoplasts extracted from leaves in CPW mineral solution and from proembryogenic masses in CPW and NEW; **(B)** Protoplasts size determined using Image J software and derived from proembryogenic masses.

The protoplast isolation was also performed starting from holm oak proembryogenic masses exposed both to CPW and NEW mineral solutions. Using this plant material, a high number of protoplasts was detected after 6h from the beginning of the experiment in both CPW and NEW solutions (**Figures 3A–D**) compared to the lower yield from leaf material, observed after 12h (**Figures 3E, F**). The highest number of protoplasts was detected (11 x 10^6^ ± 2 x 10^6^ protoplasts/ml) (**Figure 3A**) after 6 h of enzymolysis treatment in the NEW mineral solutions, with 92% ± 0.5 viability. (**Figure 3B**). Instead, in CPW mineral solution, the number of protoplasts was lower (8,8 x 10^6^ ± 5 x 10^6^ protoplasts/ml) (**Figure 3C**) with 89% ± 0.80 of viability (**Figure 3D**). In **Figure 4A** the higher yield of protoplasts extracted from embryogenic calli using both CPW and NEW mineral solution is shown, compared to the lower yield and quality of protoplasts extracted from leaves. Protoplast size was also determined by measuring the protoplast diameter using the software ImageJ v. 1.8.0; 40% of protoplasts showed a diameter between 10-20 µm followed by 30% of protoplasts with 20-30 µm size (**Figure 4B**).

### Protoplast transfection with the GFP marker gene

**Figure 5:**
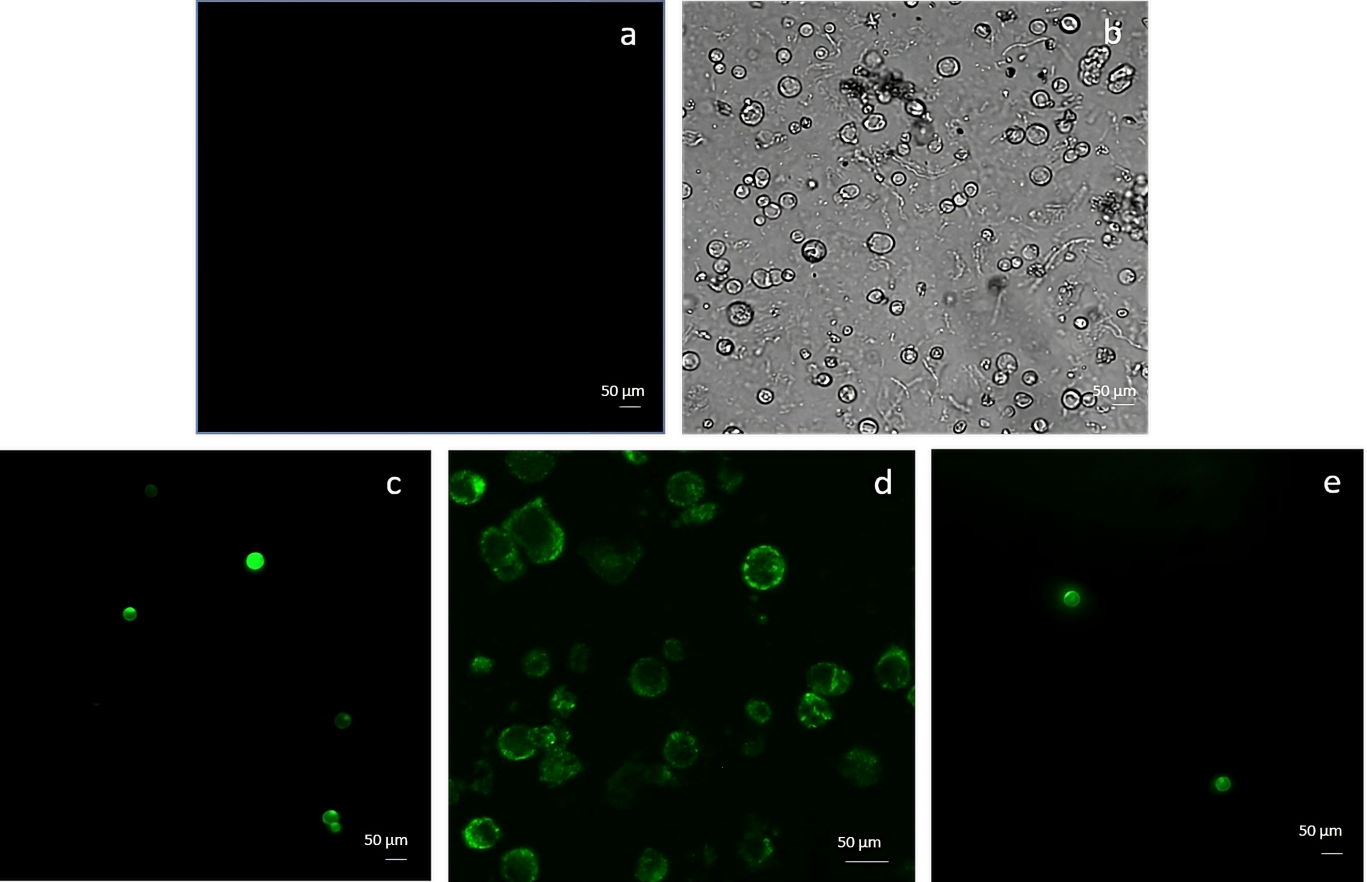
Protoplasts transfection using GFP marker gene and 10 µg plasmid. The picture represents the negative control **(A, B)** and protoplasts transfected with GFP after 12h **(C)**, 24h **(D)** and 48h **(E)** from the transfection event.

Embryogenic-derived protoplasts, isolated using the NEW mineral solution, were transfected with 10 and 20 µg of pAVA393:GFP applying PEG-mediated editing. The GFP expression was evaluated 12h, 24h, and 48h after the transfection event (**Figure 5**). After 12h, the fluorescence signal was still limited (**Figure 5C**) while at 24h the highest percentage of transfected protoplasts (62%± 8) was detected in the samples transfected with 10 µg of plasmid (**Figure 5D**). A decrease of fluorescence occurred 48h after the transfection event (**Figure 5E**) (**Supplementary Material 5A**). When protoplasts were transfected with 20 µg of pAVA393:GFP protoplasts showed lower GFP fluorescence in all the time points analysed with fluorescence values around 20% ± 5. Protoplast shape appeared intact and rounded also after 48h from the transfection process. In the negative control (vector without GFP) the absence of the fluorescence signal was confirmed (**Figures 5A, B**). GFP protoplast transfection confirmation was achieved by PCR amplification (**Supplementary Material 5B**).

### Protoplast transfection using RNPs

**Figure 6:**
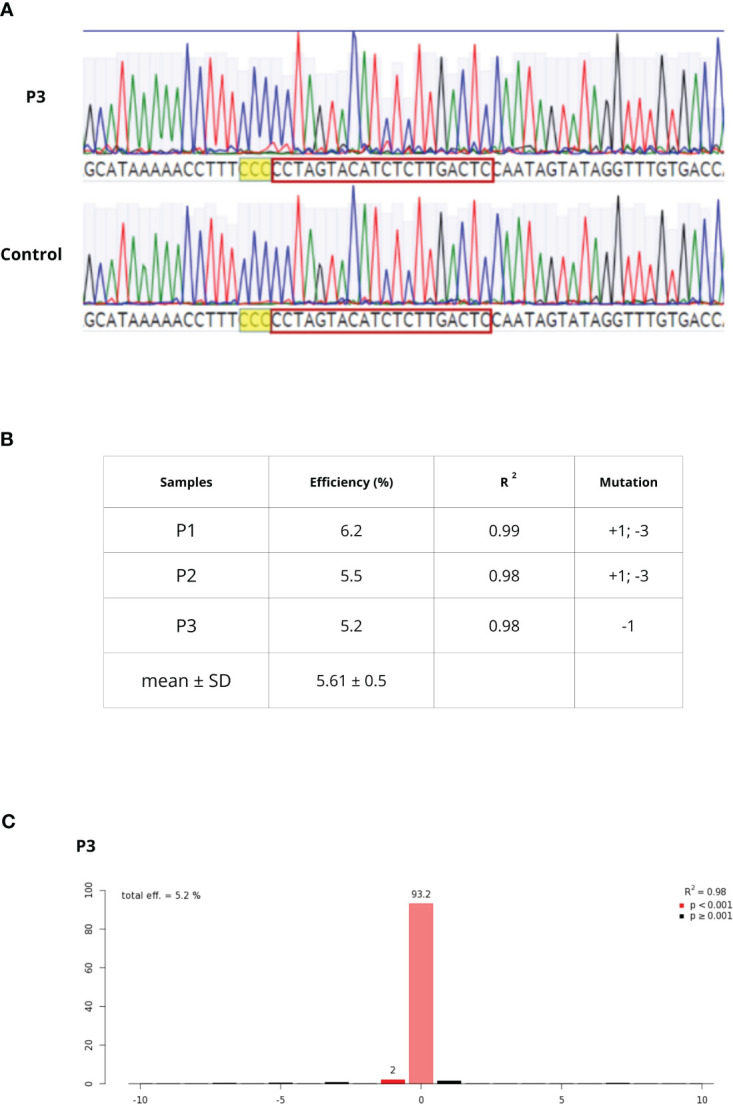
**(A)** Chromatogram alignment between WT and P3 sample. After PAM sequence (higlighted in yellow), decomposing Sanger traces made from PCR products of targeted region from WT and edited sample can be detected **(B)** editing efficiency, goodness-of-fit measure (R^2^) and mutation types in P1-P3 samples **(C)** TIDE output of the P3 sample editing efficiency and mutation.

*C. sativa pds* primers successfully amplified *Q. ilex pds* region. Through the Sanger sequencing and the MEGAX alignment, only a mismatch in the seed gRNA target region was detected between *Q. ilex pds* and *C. sativa pds* sequences; a complete identity was observed between *Q. ilex pds* and *C. mollissima pds* sequences. Once sequence conservation was established, the transfection of embryogenic calli-derived protoplasts was carried out with *pds* RNPs, in three biological replicates (P1, P2 and P3). Protoplast DNA was extracted and *pds* target region was amplified. As previously described by Michalski et al., 2023, rurified PCR products were sequenced (**Figure 6A**) and TIDE software revealed an editing efficiency of 5.6%± 0.5%. The sample P3 showed a single deletion (-1) while P1 and P2 samples showed both an insertion (+1) and a deletion (-3) (**Figures 6B, C**).

### Protoplasts regeneration

**Figure 7:**
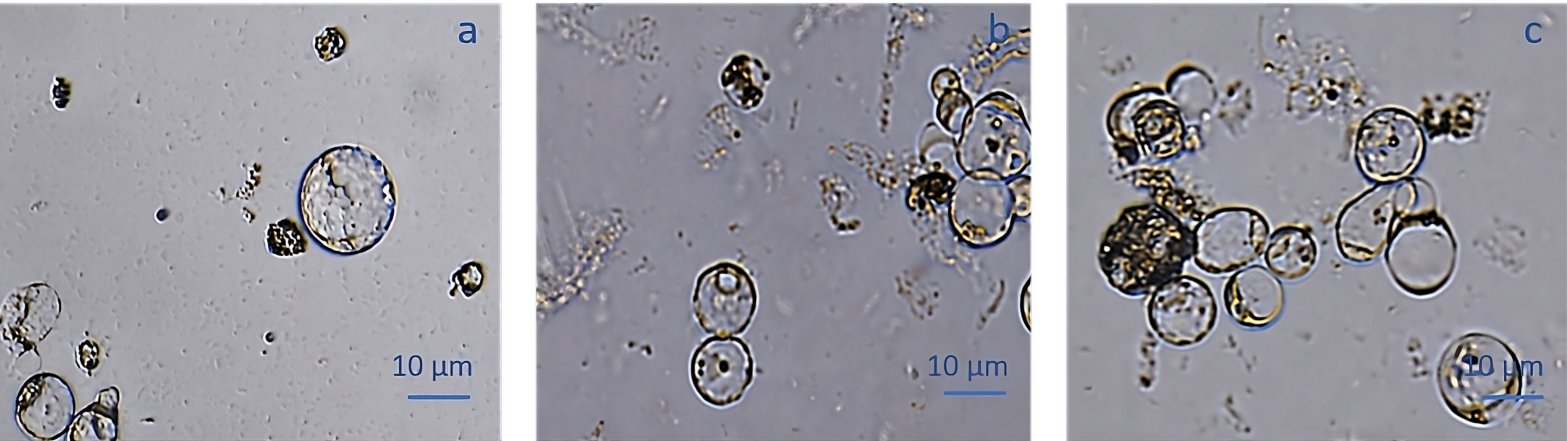
pAVA393:GFP protoplast regeneration on Q1 **(A)**, Q2 **(B)** and Q3 **(C)** media.

**Figure 8:**
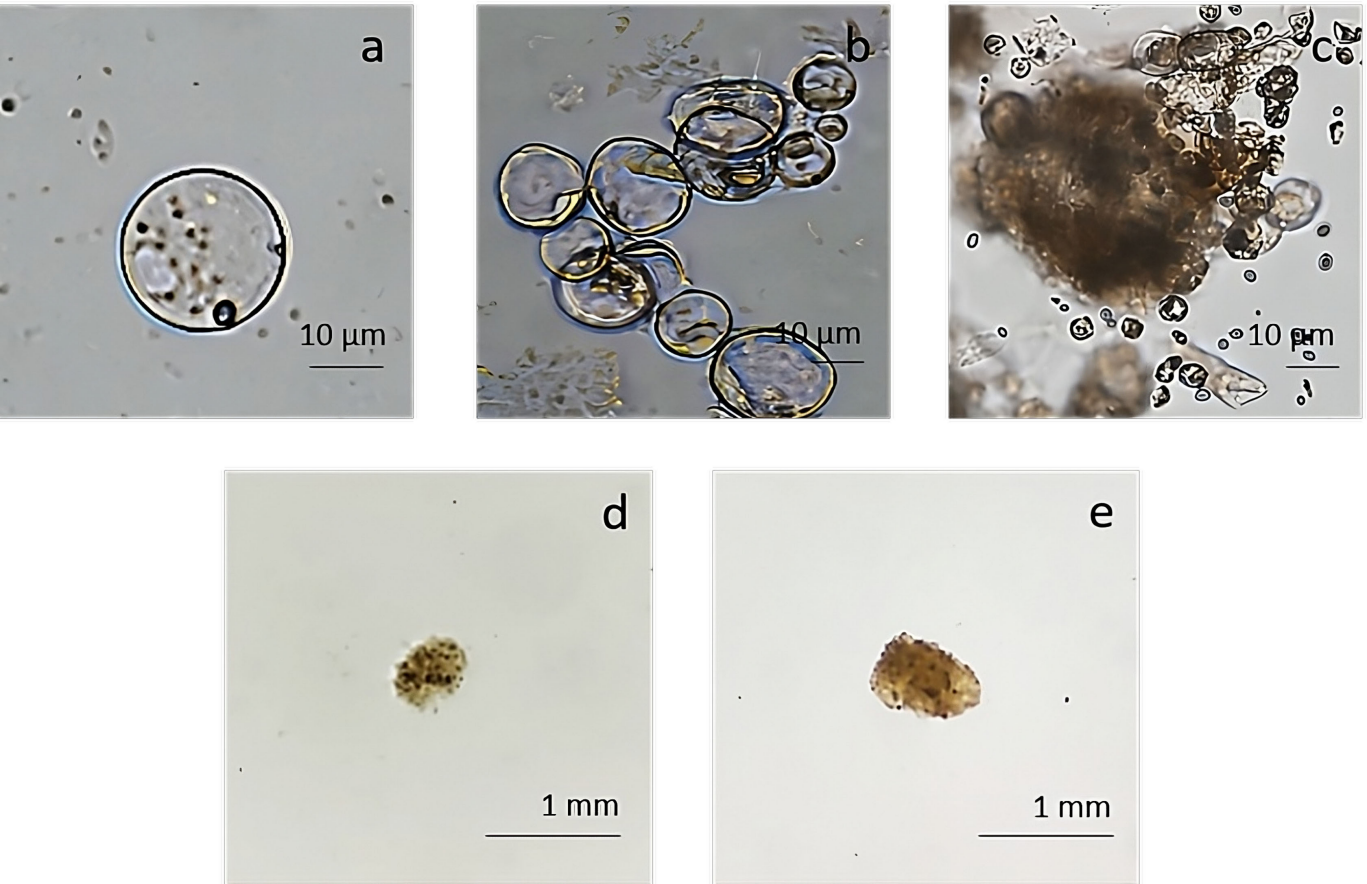
Regeneration from protoplast culture in medium Q3. **(A)** Isolated protoplasts; **(B)** first division after 5 days of culture; **(C)** microcolonies formation detected after 15 days of culture; **(D, E)** calli formation on Q3 medium after 45 days of culture.

The pAVA393:GFP protoplasts (Pavese et al., 2022)were cultured in three semi-solid regeneration media named Q1, Q2, and Q3 (**Supplementary Material 2**). The Q1 and Q2 media were not suitable for regeneration. Q3 medium consisting of MS3B medium with 1/2 concentration NH_4_NO_3_ and KNO_3_ supplemented with 0.1 mg/L BAP and 0.1 mg/L 2,4-dichlorophenoxyacetic acid (2,4D) was the most effective for protoplasts regeneration. As reported in **Figure 7**, a high number of microcolonies were visible on the Q3 compared to Q1 and Q2 after two weeks in culture. In Q3 medium different steps of protoplast regeneration were observed. In the first days protoplasts showed a perfectly spherical shape (**Figure 8A**), and the first protoplast divisions occurred after 5 days in culture (**Figure 8B**). Microcolonies formation was detected after 15 days (**Figure 8C**), whereas embryogenic calli is noted after 45 days in culture on Q3 medium (**Figures 8D, E**).

## Discussion

The holm oak is the most representative species of the Mediterranean region with a relevant ecological, economic, and social impact (Schirone et al., 2019). Oak decline syndrome is the most serious threat to holm oak as in the last decades this syndrome has produced a tremendous effect on its populations, causing significant losses. The disease is mainly caused by the oomycete *P. cinnamomi* but other pathogens and global climate change also contribute to accelerate the damages provoked by the oomycete. Currently, the use of disease-tolerant plants is considered an efficient and cost-effective alternative for managing plant diseases (Tripathi et al., 2022). In woody species, yet, long periods are necessary to apply for conventional breeding programs (Corredoira et al., 2016).

In recent years, new plant breeding techniques (NPBTs) have been developed to overcome the limitations and problems related to conventional breeding and traditional genetic transformation (Paul et al., 2021). Among these new technologies, the CRISPR/Cas9 system is widely used and is considered the most promising strategy to accelerate and improve breeding programs. Nowadays, the use of CRISPR/Cas9 in the RNPs form represents the way to cause target mutations without the use of the *A. tumefaciens* delivery, allowing the obtainment of transgene-free plants (Pavese et al., 2022). The first step in the application of this technology is obtaining a sufficient amount of high-quality protoplasts. Although protoplast isolation has now been widely defined in herbaceous plants, its development in woody species is still challenging (Zhou et al., 2021). Here we present the first report of successful demonstration of CRISPR/Cas9 RNPs mediated protoplast editing in holm oak, a very recalcitrant species to genetic transformation. In a previous report, Kuzminsky et al. (2016) obtained protoplasts from young fresh leaf tissues excised from forced shoots derived from a 15 years-old holm oak tree. In that work, protoplasts were used for comet assay technique, in order to detect DNA damage in stressed plants.

The donor material is an important factor for the successful isolation of protoplasts affecting their size, number, and viability (Reed and Bargmann, 2021). Our results clearly show that protoplast yields are higher from embryogenic tissues than from leaves. Although leaves are the most frequent source for protoplast isolation, in the last years cell suspensions or embryogenic callus are considered the best option due to the high regeneration rate showed from embryogenic cells and can be easily dissected into clumps, increasing the contact with the enzymatic solution (Davey et al., 2005; Bertini et al., 2019). On the contrary, leaves represent a material rich in phenols and lignin, which can negatively affect the activities of the cell wall degrading enzymes (Kuzminsky et al., 2016; Brandt et al., 2020; Fizree et al., 2021). High-quality protoplasts have been reported starting from embryogenic material in several woody species like coffee (Schopke et al., 1987), banana (Assani et al., 2002) and grapevine (Bertini et al., 2019).

The enzyme solution nature, concentration, and incubation conditions are critical factors for an efficient release of plant protoplasts (Davey et al., 2005). Different combinations of enzymes have been reported to degrade cell walls efficiently and they can also vary in function of donor material (Reed and Bargmann, 2021). Our results showed that 2% Cellulase R-10, 0.75% Macerozyme R-10, and 0.75% Pectolyase enzymes are the best combination for leaves, whereas in PEMs was 1% Cellulase and 0.5% Macerozyme. Hou et al. (2017) found that Pectolyase was essential for isolating toplasts from leaves of *Liriodendron* hybrid. Likewise, the presence of the Pectolyase enzyme increased the yield of protoplasts in plants such as silk tree (Rahmani et al., 2016) and *Ulmus* sp (Dorion et al., 1994). In PEMs, combination selected by us (1% Cellulase and 0.5% Macerozyme) is also the most frequently enzymatic solution mentioned for protoplasts isolation from embryogenic material (Malnoy et al., 2016; Liu et al., 2019; Pavese et al., 2022).

In the literature, the length of a digestion period is very variable, ranging from 2 to 18 h, but evaluation of enzymolysis time is an essential step (Reed and Bargmann, 2021). Prolonged enzymatic hydrolysis could cause to protoplast collapse and subsequent reduction in protoplast viability and stability, but short enzymolysis period cannot obtain good separation effect (Mukami et al., 2022). In our case, maximum protoplasts release was obtained after 12h digestion at 37°C. Similar digestion time points (10h-12h) to those tested in the present report have been applied in other hardwoods such as yellow poplar (Merkle and Sommer, 1987) and in the camphor tree (Du and Bao, 2005). Our results showed a protoplast yield and viability higher than in other woody species, like European chestnut (Pavese et al., 2022), and comparable to apple protoplasts also obtained from embryogenic calli (Malnoy et al., 2016).

The GFP marker gene is commonly used to test protoplast transfection ability (Pavese et al., 2022). GFP based selection is a good option since it does not involve the death of cells (Corredoira et al., 2016). DNA transfection can be performed by PEG, electroporation, particle bombardment, and DNA microinjection, however, the use of PEG-mediated transient editing showed higher transfection efficiency and it is cost-effective and simple in terms of releasing RNPs into the protoplasts (Shen et al., 2014; Subburaj et al., 2022). PEG has been applied to transfect protoplasts of many different plant species including woody species such as poplar (Tan et al., 2013), *Liriodendron* (Huo et al., 2017), European chestnut (Pavese et al., 2022) and banana (Zhao et al., 2022). The protoplast to plasmid DNA ratio is an essential factor influencing transfection efficiency (Burris et al., 2016), and generally the amount of plasmid DNA to be used is between 10 to 20 μg (Huo et al., 2017). The results in holm oak showed that the best transfection efficiency (62%) was achieved with 10 µg of plasmid and with GFP expression recorded 24h from the transfection process. By contrast, in *Liriodendron* (Huo et al., 2017) and banana (Zhao et al., 2022) the highest values were attained with 20 μg. Finally, our transfection values were higher than those previously obtained with protoplasts of European chestnut tree (Pavese et al., 2022) and *Cymbidium* Orchids (Ren et al., 2020).

Once the transfection ability was confirmed, the CRISPR/Cas9 RNPs were delivered to holm oak protoplasts. We used the crRNA designed on the chestnut *pds*, once confirmed the sequence homology between both species. These two species belong to *Fagales* order, and they are closely related as previously reported in Pavese et al. (2021b). This allows the possibility of technology transfer in related species. The editing efficiency varying from 5.2 to 6.2% achieved in our paper was lower than the results reported using RNPs in other species such as potato (9-25%, Andersson et al., 2018), Arabidopsis (16%), tobacco (44%) and rice (19%) (Woo et al., 2015). By contrast, similar low editing frequencies for protoplasts were reported in woody species such as apple (0.5-6.9%), grape (0.1%) (Malnoy et al., 2016) and Cavendish banana (0.19-0.92%) (Wu et al., 2020).

After culture in semisolid medium, microcalli became visible after 6 weeks but somatic embryos were not obtained. The regeneration step is the major bottleneck for woody species (Corredoira et al., 2019), in particular starting from protoplasts (Attre et al., 1989; Papadakis and Roubelakis-Angelakis, 2002). This is particularly relevant for holm oak due to its high recalcitrance to *in vitro* culture (Martinez et al., 2017). Among the factors influencing the regeneration from protoplasts, auxins and cytokinins play a critical role in the regeneration step (Sandgrind et al., 2021). In our work, the highest callus induction rate was obtained by using a combination of BAP and 2,4D. It has been demonstrated that the auxin 2,4-D is essential for the formation of the cell wall and the initial protoplast growth as previously observed in other species (Shi et al., 2016; Tu et al., 2023). Although we failed in somatic embryo formation and plantlet regeneration under the procedure defined by us microcolonies and embryogenic callus formation were achieved and it provides a base for further optimization.

## Conclusions

In the present work, we set up the first protocol for protoplast isolation both from *in vitro* leaves and proembryogenic masses of holm oak, a recalcitrant species. Embryogenic masses represent the most interesting matrix for greater quantity and quality protoplasts. In addition, we demonstrated that the protoplasts produced with our protocol are competent for the DNA transfection. Interestingly, the CRISPR/Cas9 machineryusing RNPs was successfully applied for the first time in holm oak and the first transgene-free protoplasts were obtained and submitted to regeneration. Future work will be aimed to optimize the regeneration protocol from protoplasts. The present RNP-based method is highly promising to enhance the holm oak breeding. Holm oak is susceptible to *P. cinnamomi* and susceptibility genes *pmr4* and *dmr6*, already detected in chestnut, can be interesting targets for gene editing events (Pavese et al., 2021b).

## Data availability statement

The original contributions presented in the study are included in the article/**Supplementary Material**. Further inquiries can be directed to the corresponding author.

## Author contributions

VP: Conceptualizazion, Data curation, Investigation, Writing – original draft, Writing - review & editing. AM: Conceptualizazion, Data curation, Investigation, Supervision, Writing - review & editing. AMM: Investigation, Writing - review & editing. LAM: Investigation, Writing - review & editing. MTM: Investigation, Supervision, Writing - review & editing. DTM: Conceptualizazion, Supervision, Writing - review & editing. RB: Conceptualization, Supervision, Writing - review & editing. EC: Conceptualization, Data curation, Investigation, Supervision, Writing – original draft, Writing - review & editing.
