# Supplementary material for: Advances in Quercus ilex L. breeding: the CRISPR/Cas9 technology via ribonucleoproteins
Source: Front Plant Sci. 2024 Feb 19;15:1323390. doi: 10.3389/fpls.2024.1323390 (PMC10910054; doi:10.3389/fpls.2024.1323390)
Supplement: Supplementary file 1 [file DataSheet_1.zip › Supplementary Material 1.docx]

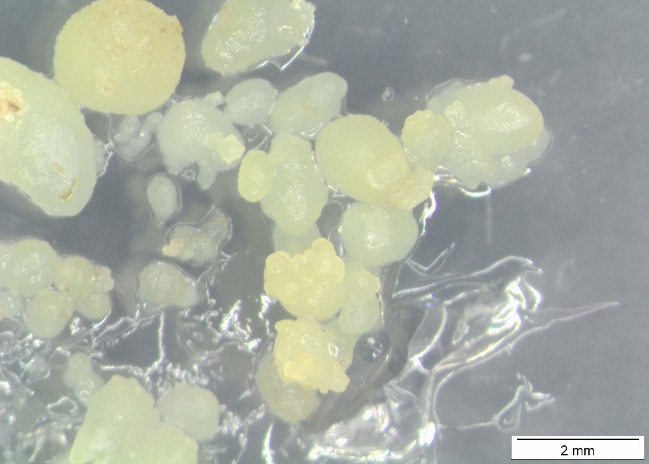


**b**


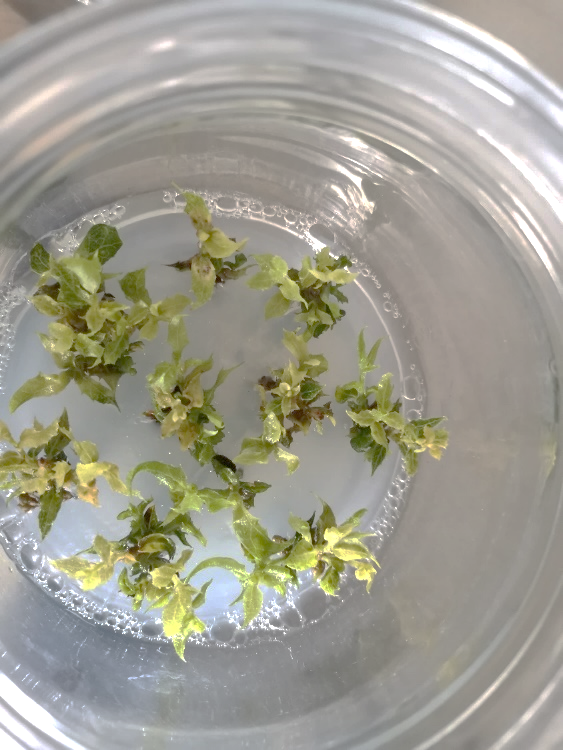


**a**

**Supplementary Material 1.** Aspect of explants used as initial material to obtain protoplasts in holm oak. a) Axillary shoot cultures used to obtain leaves. b) Proembryogenic masses.
