# Supplementary material for: Advances in Quercus ilex L. breeding: the CRISPR/Cas9 technology via ribonucleoproteins
Source: Front Plant Sci. 2024 Feb 19;15:1323390. doi: 10.3389/fpls.2024.1323390 (PMC10910054; doi:10.3389/fpls.2024.1323390)
Supplement: Supplementary file 1 [file DataSheet_1.zip › Supplementary Material 2.docx]

**Supplementary Material 2.**

Solutions and media used for protoplasts isolation and regeneration.

***CPW solution***

# KH_2_PO_4:_ Monopotassium phosphate; KNO_3:_ Potassium nitrate; CaCl_2_ x 2 H_2_O: Calcium chloride dihydrate; MgSO_3_ x 7 H_2_O: Magnesium sulfate Heptahydrate; KI: Potassium iodide; CuSO_4_ x 5 H_2_O: Copper(II) chloride dihydrate; BSA: Bovine serum albumine.

| KH_2_PO_4_ | 0.2 mM |
| --- | --- |
| KNO_3_ | 1 mM |
| CaCl2 x 2 H_2_O | 10.1 mM |
| MgSO_3_ x 7 H_2_O | 1 mM |
| KI | 0.96 μM |
| CuSO_4_ x 5 H_2_O | 0.16 μM |
| D-Mannitol | 0.5 M |
| BSA | 0.1% |

***NEW solution***

MES: 2-[N-morpholino]-ethanesulfonic acid; CaCl_2_: calcium chloride; KCl: potassium chloride

| MES | 20 mM |
| --- | --- |
| D-Mannitol | 0.5 M |
| KCl | 20 mM |
| CaCl_2_ | 10 mM |

***Washing solution (WS)***

NaCl: sodium chloride;

| Glucose | 5 mM |
| --- | --- |
| MES | 2 mM |
| NaCl | 154 mM |
| CaCl_2_ | 125 mM |
| KCl | 5 mM |

***MMG solution***

MgCl_2_: magnesium chloride

| D-Mannitol | 0,5 M |
| --- | --- |
| MES | 4 mM |
| MgCl_2_ | 15 mM |

***Q1 medium***

BAP: 6-benzyladeninepurine; AgNO_3_: Silver nitrate

| Woody Plant Medium | 2.46 g/L |
| --- | --- |
| BAP | 0.2 mg/L |
| Cefotaxime | 0.1 mg/L |
| Carbenicellin | 0.1 mg/L |
| AgNO_3_ | 0.2 mg/L |
| Sucrose | 5 g/L |
| D-Mannitol | 90 g/L |
| Plant agar | 6 g/L |

***Q2 medium***

*MS3B: Murashige and Skoog* with half concentration of NH_4_NO_3_ and KNO_3_*;* 2,4-D: 2,4-dichlorophenoxyacetic acid.

| MS3B | 2.6 g/L |
| --- | --- |
| BAP | 0.1 mg/L |
| 2,4-D | 0.01 mg/L |
| Sucrose | 5 g/L |
| D-Mannitol | 90 g/L |
| Plant agar | 6 g/L |
|  |  |

***Q3 medium***

| MS3B | 2.6 g/L |
| --- | --- |
| BAP | 0.1 mg/L |
| 2,4-D | 0.1 mg/L |
| Sucrose | 5 g/L |
| D-Mannitol | 90 g/L |
| Plant agar | 6 g/L |
