# Supplementary material for: Advances in Quercus ilex L. breeding: the CRISPR/Cas9 technology via ribonucleoproteins
Source: Front Plant Sci. 2024 Feb 19;15:1323390. doi: 10.3389/fpls.2024.1323390 (PMC10910054; doi:10.3389/fpls.2024.1323390)

**Supplementary Material 3.** Schematic overview of the materials and procedures used in the protoplast extraction protocol.


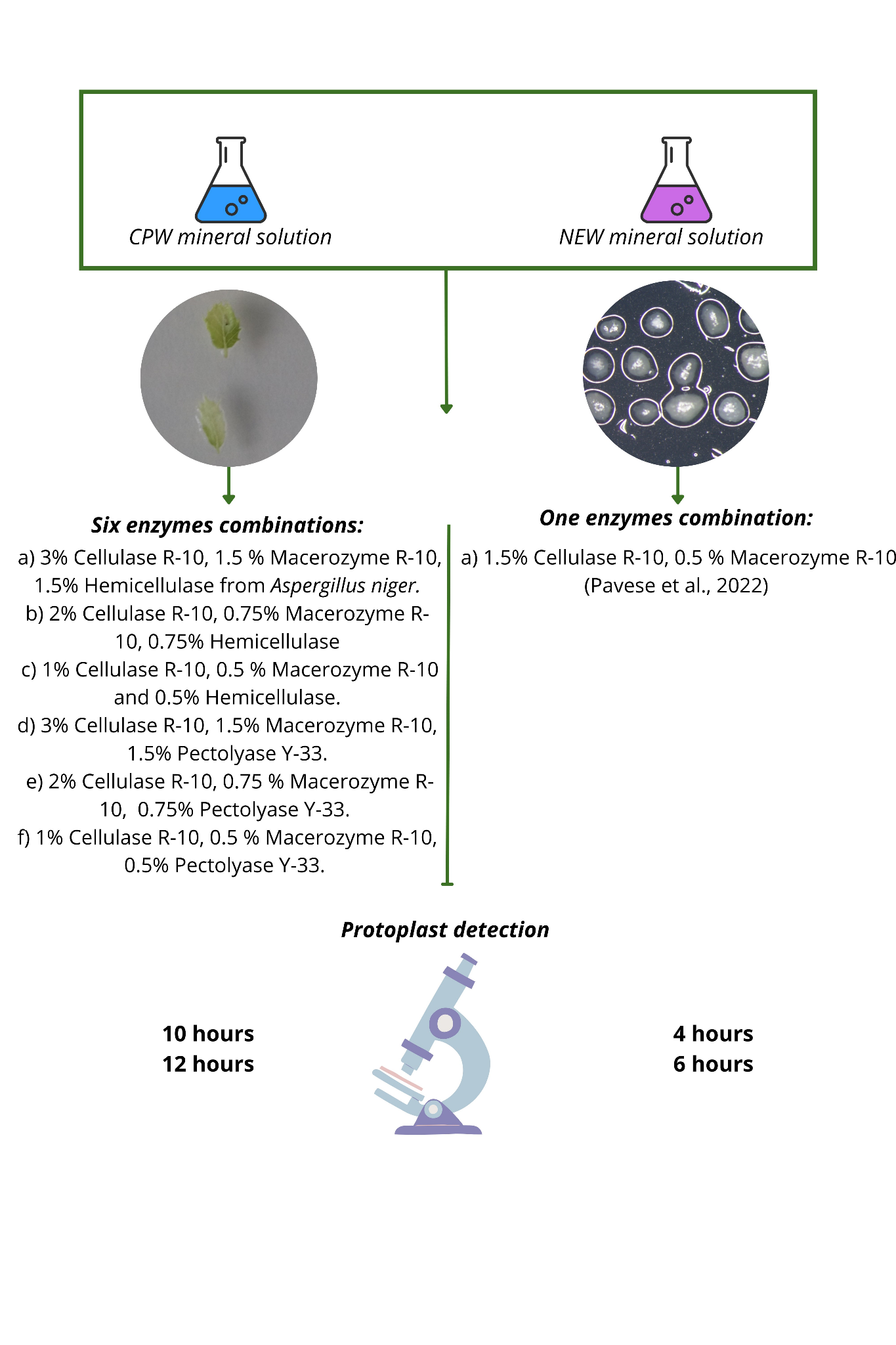

Supplement: Supplementary file 1 [file DataSheet_1.zip › Supplementary Material 3.docx]
