# Supplementary material for: Advances in Quercus ilex L. breeding: the CRISPR/Cas9 technology via ribonucleoproteins
Source: Front Plant Sci. 2024 Feb 19;15:1323390. doi: 10.3389/fpls.2024.1323390 (PMC10910054; doi:10.3389/fpls.2024.1323390)
Supplement: Supplementary file 1 [file DataSheet_1.zip › Supplementary Material 4.docx]

**Supplementary Material 4.**

Quercus spp. *pds* sequences available on NCBI database (<https://www.ncbi.nlm.nih.gov/>) and reference *C. sativa* *pds* sequence used by Pavese et al., 2022. A Clustal W alignment (<https://www.ebi.ac.uk/Tools/msa/clustalo/>) was performed to detect gRNA homology, indicated with a red line between *Castanea* and *Quercus* species. The blue arrows evidenced the *Quercus* gRNA mismatch.

>*Quercus suber* ATGACCATTGGTGGGTTTGTTTCGGCTGCAAACTTGAGCTGCCAAAGTACTTTGACAGGAAATCAAACTCTGGGATGTGGGTTTCTTAATAATTCAGTGAAAACCAATGCATTAGCATTTGGAGGTTGTGAATCCATGGGTCATAGTTTGAGAATTCCACATACAAAGGCTATTAGATTGAGGCCGAGGAAGGGTGTCTCTCCTTTGCAGGTAGTATGTATGGACTTCCCAAGACCAGAGCTTGAGAATACTGTTAATTTCTTAGAGGCTGCTTATTTGTCTTCTTCCTTTCGTGCATCTGCTCGTCCATCTAAACCCCTAACAGTTGTAATTGCTGGTGCAGGTTTGGCTGGTTTGTCTACTGCAAAGTATTTGGCAGATGCTGGTCACAAACCTATACTATTGGAGTCAAGAGATGTA CTAGGAGGAAAGGTGGCTGCATGGAAAGATGACGATGGAGACTGGTATGAGACTGGATTACATATATTCTTTGGGGCTTACCCAAATGTGCAGAATCTGTTCGGAGAACTTGGTATTGATGATCGGTTGCAATGGAAGGAACATTCTATGATTTTTGCAATGCCAAATAAGCCGGGAGAGTTCAGCCGATTTGATTTTCCTGAAGTTCTTCCTGCACCATTAAATGGAATATGGGCTATCTTGAAGAACAATGAGATGCTGACTTGGCCAGATAAAGTCAAGTTTGCGATTGGACTCTTGCCGGCAATGCTTGGTGGACAGGCTTATGTTGAAGCTCAAGATGGTTTAACTGTTAAAGAGTGGATGAGAAAGCAGGGAGTACCTGATCGTGTAACTGATGAGGTATTTGTAGCCATGTCAAAGGCGCTAAACTTCATTAACCCCGATGAACTTTCAATGCAATGCATATTGATTGCTTTGAATAGGTTTCTTCAGGAGAAGAATGGTTCCAAGATGGCTTTCTTGGATGGTAATCCCCCAGAGAGACTCTGTATGCCAATTGTTGATCATATTCAATCACTAGGTGGTGAAGTAAGACTAAATTCGAGAATACAAAAAATCGAGCTAAATAATGATGGAACAGTGAAAAGCTTTTTACTGAATAATGGGAACATGATTGAAGGAGATGCTTATGTATTTGCTACTCCAGTTGATATCCTGAAGCTTCTTTTGCCGGAAAACTGGAAAGAGATTCCATATTTCCAGAGATTAAAGAAATTAGTTGGAGTTCCAGTTATTAATGTCCACATATGGTTTGACAGAAAACTGAAGAACACCTATGATCACCTACTGTTTAGCAGAAGTCCACTTCTCAGTGTGTATGCTGACATGTCAGTAACATGTAAGGAATATTACAACCCAAACCAATCTATGCTGGAGTTGGTTTTTGCGCCTGCAGAAGAATGGATTTCACGCAGTGACTCAGACATTATTGACGCTACAATGAATGAACTTGCAAGACTCTTTCCTGATGAAATTTCCACGGATCAAAGCAAAGCAAAGATTGTGAAGTACCATGTTGTTAAAACACCAAGGTCTGTTTACAAAACTGTCCCAGACTGTGAACCTTGCCGTCCCTTACAAAGATCTCCTATTGAGGGGTTTTACTTAGCTGGTGACTACACAAAACAAAAATATTTGGCTTCAATGGAAGGTGCTGTTCTGTCAGGAAAGCTTTGTGCTCAGGCTATTGTACAGGATTATGAGTTGCTTATAGCTCGGGGGCAAACAAGGTTGGCTCAAGCAAGTGTTTATTGA

>*Quercus lobata* ATGACCATTGGTGGGTTTGTTTCGGCTGCAAACTTGAGCTGCCAAAGTACTTTGACAGGAAATCAAACTCTGAGATGTGGGTTTCTTAATAATTCAGTGAAAACCAATGCATTAGCATTTGGAGGTTGTGAATCCATGGGCCATAGTTTGAGAATTCCACATACAAAGGCTATTAGATTGAGGCCGAGGAAGGGTGTCTCTCCTTTGCAGGTAGTATGTATGGACTTTCCAAGACCAGAGCTTGAGAATACTGTTAATTTCTTAGAGGCTGCTTATTTGTCTTCTTCCTTTCGTGCATCTGCTCGTCCATCTAAACCCCTAACAGTTGTAATTGCTGGTGCAGGTTTGGCTGGTTTGTCTACTGCAAAGTATTTGGCAGATGCTGGTCACAAACCTATACTATTGGAGTCAAGAGATGTACTAGGAGGAAAGGTGGCTGCATGGAAAGATAACGATGGAGACTGGTATGAGACTGGATTACATATATTCTTTGGGGCTTACCCAAATGTGCAGAATCTGTTCGGAGAACTTGGTATTGATGATCGGTTGCAATGGAAGGAGCATTCTATGATTTTTGCAATGCCAAATAAGCCGGGAGAGTTCAGCCGATTTGATTTTCCTGAAGTTCTTCCTGCACCATTAAATGGAATATGGGCTATCTTGAAGAACAATGAGATGCTGACTTGGCCAGATAAAGTCAAGTTTGCGATTGGACTCTTGCCAGCAATGCTTGGTGGACAGGCTTATGTTGAAGCTCAAGATGGTTTAACTGTTAAAGAGTGGATGAGAAAGCAGGGAGTACCTGATCGTGTAACTGATGAGGTATTTGTAGCCATGTCA AAGGCGCTAAACTTCATTAACCCCGATGAACTTTCAATGCAATGCATATTGATTGCTTTGAATAGGTTTCTTCAGGAGAAGAATGGTTCCAAGATGGCTTTCTTGGATGGTAATCCCCCAGAGAGACTCTGTATGCCAATTGTTGATCATATTCAATCACTAGGTGGTGAAGTAAGACTAAATTCAAGAATACAAAAAATCGAGCTAAATAATGATGGAACAGTGAAAAGCTTTTTACTGAATAATGGGAACATGATTGAAGGAGATGCTTATGTATTTGCTACTCCGGTTGATATCCTGAAGCTTCTTTTGCCGGAAAACTGGAAAGAGATTCCATATTTCCAGAGATTAAAGAAATTAGTTGGAGTTCCAGTTATTAATGTCCACATATGGTTTGACAGAAAACTGAAGAACACCTATGATCACCTACTGTTTAGCAGAAGTCCACTTCTCAGTGTGTATGCTGACATGTCAGTAACATGTAAGGAATATTACAACCCAAACCAATCTATGCTGGAGTTGGTTTTTGCGCCTGCAGAAGAATGGATTTCACGCAGTGACTCAGACATTATTGACGCTACAATGAATGAACTTGCAAGACTCTTTCCCGATGAAATTTCCACGGATCAAAGCAAAGCAAAGATTGTGAAGTACCATGTTGTTAAAACACCAAGGTCTGTTTACAAAACTGTCCCAGACTGTGAACCTTGCCGTCCCTTACAAAGATCTCCTATTGAGGGGTTTTACTTAGCTGGTGACTACACAAAACAAAAATATTTGGCTTCAATGGAAGGTGCTGTTCTGTCAGGAAAGCTTTGTGCTCAGGCTATTGTACAGGATTATGAGTTGCTTATAGCTCGGGGGCAAACAAGGTTGGCTCAAGCAAGTGTTTATTGA

>*Quercus robur*

ATGACCATTGGTGGGTTTGTTTCGGCTGCAAACTTGAGCTGCCAAAGTACTTTGACAGGAAATCAAACTCTAAGATGTGGGTTTCTTAATAATTCAGTGAAAACCAATGCATTAGCATTTGGAGGTTGTGAATCCATGGGTCATAGTTTGAGAATTCCACTTACAAAGGCTATTAGATTGAGGCCGAGGAAGGGTGTCTCTCCTTTGCAGGTAGTATGTATGGACTTTCCAAGACCAGAGCTTGAGAATACTGTTAATTTCTTAGAGGCTGCTTATTTGTCTTCTTCCTTTCGTGCATCTGCTCGTCCATCTAAACCCCTAACAGTTGTAATTGCTGGTGCAGGTTTGGCTGGTTTGTCTACTGCAAAGTATTTGGCAGATGCTGGTCACAAACCTATACTATTGGAGTCAAGAGATGTACTAGGAGGAAAGGTGGCTGCATGGAAAGATGACGATGGAGACTGGTATGAGACTGGATTACATATATTCTTTGGGGCTTACCCAAATGTGCAGAATCTGTTCGGAGAACTTGGTATTGATGATCGGTTGCAATGGAAGGAACATTCTATGATTTTTGCAATGCCAAATAAGCCGGGAGAGTTCAGCCGATTTGATTTTCCTGAAGTTCTTCCTGCACCATTAAATGGAATATGGGCTATCTTGAAGAACAATGAGATGCTGACTTGGCCAGATAAAGTCAAGTTTGCGATTGGACTCTTGCCGGCAATGCTTGGTGGACAGGCTTATGTTGAAGCTCAAGATGGTTTAACTGTTAAAGAGTGGATGAGAAAGCAGGGAGTACCTGATCGTGTAACTGATGAGGTTTTTGTAGCCATGTCA

AAGGCGCTAAACTTCATTAACCCCGATGAACTTTCAATGCAATGCATATTGATTGCTTTGAATAGGTTTCTTCAGGAGAAGAATGGTTCCAAGATGGCTTTCTTGGATGGTAATCCCCCAGAGAGACTCTGTATGCCAATTGTTGATCATATTCAATCACTAGGTGGTGAAGTAAGACTAAATTCGAGAATACAAAAAATCGAGCTAAATAATGATGGAACAGTGAAAAGCTTTTTACTGAATAATGGGAACATGATTGAAGGAGATGCTTATGTATTTGCTACTCCAGTTGATATCCTGAAGCTTCTTTTGCCGGAAAACTGGAAAGAGATTCCATATTTCCAGAGATTAAAGAAATTAGTTGGAGTTCCAGTTATTAATGTCCACATATGGTTTGACAGAAAACTGAAGAACACCTATGATCACCTACTGTTTAGCAGAAGTCCACTTCTCAGTGTGTATGCTGACATGTCAGTAACATGTAAGGAATATTACAACCCTAATCAATCTATGCTGGAGTTGGTTTTTGCGCCTGCAGAAGAATGGATTTCACGCAGTGACTCAGACATTATTGACGCTACAATGAATGAACTTGCAAGACTCTTTCCCGATGAAATTTCCACGGATCAAAGCAAAGCAAAGATTGTGAAGTACCATGTTGTTAAAACACCAAGGTCTGTTTACAAAACTGTCCCAGACTGTGAACCTTGCCGTCCCTTACAAAGATCTCCTATTGAGGGGTTTTACTTAGCTGGTGACTACACAAAACAAAAATATTTGGCTTCAATGGAAGGTGCTGTTCTGTCAGGAAAGCTTTGTGCTCAAGCTATTGTACAGGATCATGAGTTGCTTATAGCTCGGGGGCAAACAAGGTTGGCTCAAGCAAGTGTTTATTGA

>*Castanea sativa* GACAGGAAATCAAACTCTGAGATGTGGGTTTCTTAATAATTCGGTGAAAACCAATGCATTAGCATTTGGAGGTTGTGAATCCATGGGTCATATTTtGAGAATTCCACATACAAAGGCTATTAGATTGAGGCCGAGGAAGGGTGTCTCTCCTTTGCAGGTAGTATGTATGGACTTTCCAAGACCAGAGCTTGAGAATACTGTTAATTTCTTAGAGGCTGCTTATTTGTCTTCTTCCCTCCGTGCATCTGCTCGTCCATCTAAaCCCCTAACAGTTGTAATTGCTGGTGCAGGTTTGGCTGGTTTGTCTACTGCAAAGTATTTGGCAGATGCTGGTCACAAACCTATACTATTGGAGTCAAGAGATGTGCTAggaGGAaaGGTGGCTGCATGGAAAGATGACGATGGAGActGGTATGAGACTGGATTACATATATTCTTTGGGGCTTACCCAAATGTGCAGAATCTTTTtGGAGAACTTGGTATTGATGATCGGTTGCAATGGAaGGAACATTCTATGATTTTtGCAATGCCAAATAAGCCGGGAGAGTTCAGCCGATTTGATTTTCCTGAAGTTCTTCCTGCACCATTAAATGGAATATGGGCTATTTTGAAGAACAATGAGATGCTGACTTGGCCAGATAAAGTCAAGTTTGCAATTGGACTCTTGCCAGCAATGCTTGGTGGACAGGCTTATGTTGAAGCACAAGATGGTTTAACTGTTAAAGAGTGGATGAGAAAGCAGGGAGTACCTGATCGTGTAACTGATGAGGTGTTTGTAGCCATGTCAAAGGCGCTAAACTTCATTAACCCTGATGAACTTTCAATGCAATGCATATTGATTGCTTTGAATAGGTTTCTTCAGGAGAAGAATGGTTCCAAGATGGCTTTCTTGGATGGTAATCCCCCAGAGAGACTCTGTATGCCAATTGTTGATCATATTCAATCACTCGGcGGTGAAGTAAGACTGAATTCGAGAATACAAAAAATCGAGCTAAATAATGATGGAACAGTGAAAAGCTTTTTACtGAATAATGGGAACATGATTGAAGGAGATGCTTATGTATTtgCTaCTCCAGTTGATATCCTGAAGCTTCTTTTGCCGGAAAACTGGAAAGAGATTCCATATTTtCAGAGATTAAAGAAATTAGTTGGAGTTCCAGTTATTAATGTCCACATaTGGTTTGACAGAAAACTGAAGAACACCTATGATCACCTACTGTTTAGCAGAAGTCcACTTCTCAGTGTGTATGCTGACATGTCACTAACgTGTAAGGAATATTACAACCCAAACcAATCTATGCTGGAGTTGGTTTTTGCgCCTGCAGAAGAATGGATTTCATGCAGTGACTCAGACATTATTGACGCTACAATGAATGAACTTGCAAGACTCTTTCCCGATGAAATTTCCACGgATCAAAGCAAAGCAAAGATTGTGAAGTACCATGTTGTTAAAACACCAAGGTCTGTTTACAAAATTGT CCCAGACTGTGAACCTTGC


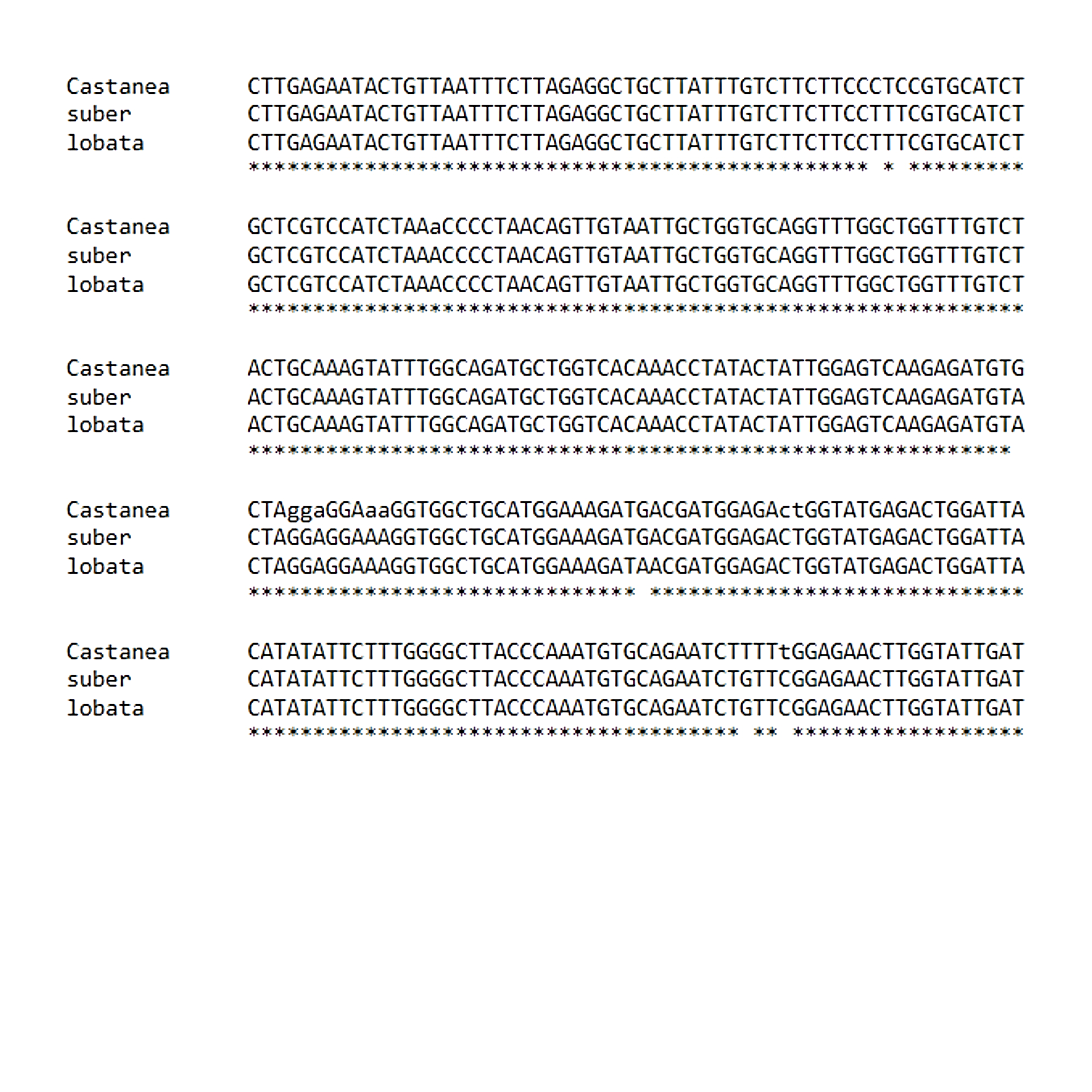


*C. sativa*

*Q. suber*

*Q. lobata*
