# Supplementary material for: Advances in Quercus ilex L. breeding: the CRISPR/Cas9 technology via ribonucleoproteins
Source: Front Plant Sci. 2024 Feb 19;15:1323390. doi: 10.3389/fpls.2024.1323390 (PMC10910054; doi:10.3389/fpls.2024.1323390)
Supplement: Supplementary file 1 [file DataSheet_1.zip › Supplementary Material 5.docx]

**Supplementary Material 5.**

A) Transfection efficiency evaluated 12, 24 and 48 hours from the protoplast transfection with pAVA393:GFP. Three biological replicates were analysed. Different letters associated with the set of means indicate a significant difference based on Tukey’s HSD test (p ≤ 0.05).

B) PCR-mediated confirmation of the transfection evaluated 12, 24 and 48 hours from the protoplast transfection with pAVA393:GFP, Plasmid specific primers (above) and housekeeping Actin primers (below) were used. Three biological replicates were analysed. Ladder 100-300 bp.


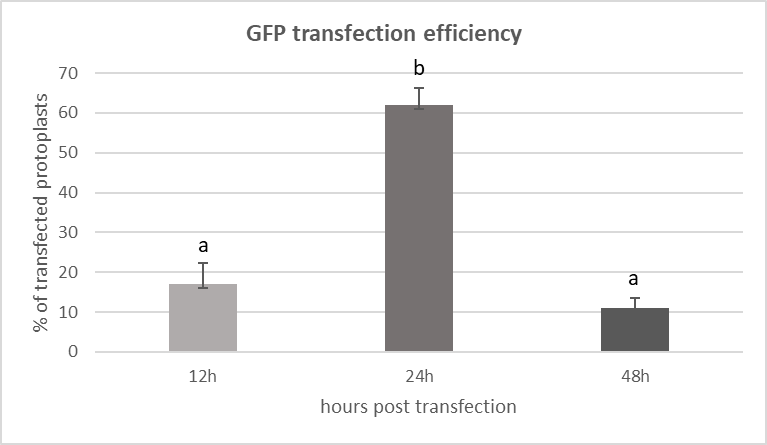


A


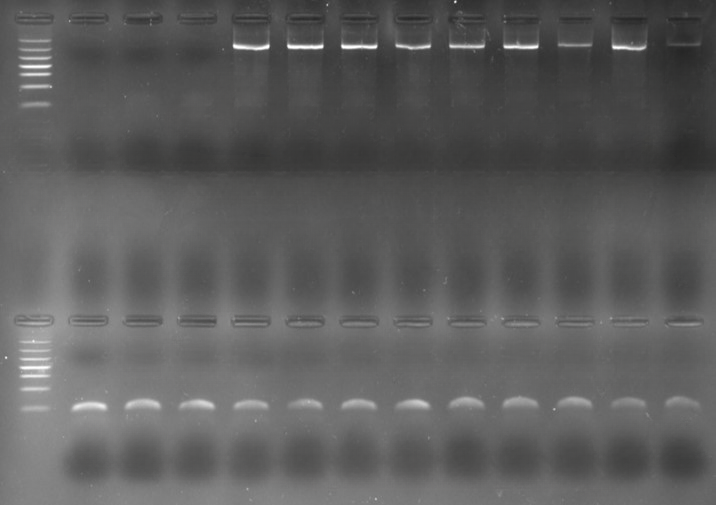


Ladder

Control_1

Control_2

Control_3

12h_1

12h_2

12h_3

24h_1

24h_2

24h_3

48h_1

48h_2

48h_3

B

Control_3
